# MPS-Net: Multi-Point Supervised Network for CT Image Segmentation of COVID-19

**DOI:** 10.1109/ACCESS.2021.3067047

**Published:** 2021-03-19

**Authors:** Hong-Yang Pei, Dan Yang, Guo-Ru Liu, Tian Lu

**Affiliations:** Key Laboratory of Infrared Optoelectric Materials and Micro-Nano DevicesNortheastern University 27817 Shenyang 110819 China; Key Laboratory of Data Analytics and Optimization for Smart Industry, Ministry of EducationNortheastern University 27817 Shenyang 110819 China; College of Information Science and EngineeringNortheastern University 27817 Shenyang 110819 China

**Keywords:** COVID-19, CT, U-Nnet, MPS-Net

## Abstract

The new coronavirus, which has become a global pandemic, has confirmed more than 88 million cases worldwide since the first case was recorded in December 2019, causing over 1.9 million deaths. Since COIVD-19 lesions have clear imaging features on CT images, it is suitable for the auxiliary diagnosis and treatment of COVID-19. Deep learning can be used to segment the lesions areas of COVID-19 in CT images to help monitor the epidemic situation. In this paper, we propose a multi-point supervision network (MPS-Net) for segmentation of COVID-19 lung infection CT image lesions to solve the problem of a variety of lesion shapes and areas. A multi-scale feature extraction structure, a sieve connection structure (SC), a multi-scale input structure and a multi-point supervised training structure were implemented into MPS-Net. In order to increase the ability to segment various lesion areas of different sizes, the multi-scale feature extraction structure and the sieve connection structure will use different sizes of receptive fields to extract feature maps of various scales. The multi-scale input structure is used to minimize the edge loss caused by the convolution process. In order to improve the accuracy of segmentation, we propose a multi-point supervision training structure to extract supervision signals from different up-sampling points on the network. Experimental results showed that the dice similarity coefficient (DSC), sensitivity, specificity and IOU of the segmentation results of our model are 0.8325, 0.8406, 09988 and 0.742, respectively. The experimental results demonstrated that the network proposed in this paper can effectively segment COVID-19 infection on CT images. It can be used to assist the diagnosis and treatment of new coronary pneumonia.

## I. Introduction

A novel Coronavirus Disease (COVID-19) has spread rapidly around the world since the beginning of 2020. It was declared a pandemic by the World Health Organization (WHO) on 11 March of the same year, treating it as a public health emergency of international concern (PHEIC) [1]–[4]. As of January 12, 2021, COIVD-19 has caused more than 88 million infections and more than 1.9 million deaths [5]. The number continues to rise every day worldwide.

Early detection, quarantine and effective treatment are the best ways to slow and stop the rapid spread of the virus. In clinical practice, reverse transcription polymerase chain reaction (RT-PCR) is the gold standard for the definite diagnosis of COVID-19 infection [6]. But because of the limited diagnostic equipment available, many countries can only test a limited number of their citizens for COVID-19 [7], [8]. In addition, RT-PCR detection has a high false-negative rate and fails to identify the degree of infection. Therefore, this method is difficult to carry out targeted treatment [9].

**FIGURE 1.**
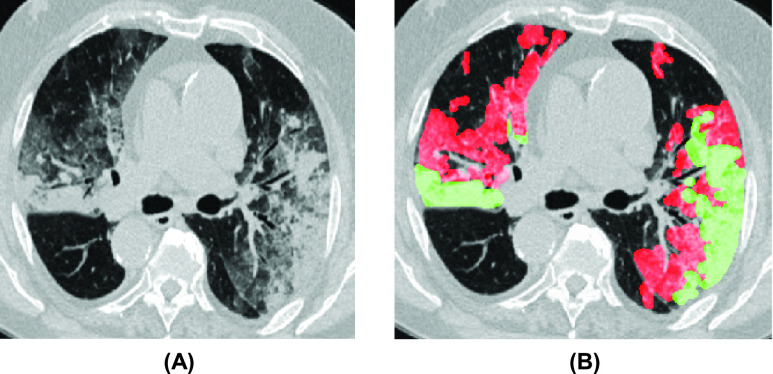
Examples of CT(B) images and original CT images (A) of COVID-19 lesions. The red and green masks represent GGO and solids, respectively. This image is from [11].

The chest computed tomography (CT) provides a noninvasive and effective method for the detection of viral pneumonia. CT images can not only help determine whether a patient is infected with COVID-19, but also show the evolution of pneumonia in the infected area of the lung at different times. For example, typical radiological features of almost all COVID-19 patients on chest CT include ground-glass opacity (GGO) in the early stage, and pulmonary consolidation in the late stage [10], as shown in Fig.1 In clinical applications, segmentation of different organs and lesions from chest CT slices can provide important information for doctors to diagnose and quantify pulmonary diseases [15]. However, manual segmentation of lesions on CT images requires extensive experience of the radiologist. Due to the increasing number of patients, using CT frequently has placed a heavy burden on the radiology department. Therefore, there is an urgent need to find a low-cost, rapid tool to effectively detect and diagnose COVID-19.

Deep learning has been widely applied in autonomous driving, facial recognition and medical image processing in recent years [12]–[14]. Convolutional Neural Networks (CNNs) is one of the most representative deep learning technologies, which has been used to process medical images in many researches. At present, many common deep learning networks are used for image segmentation including FCN [16], SegNet [17], U-Net [18], etc. U-Net and the modified U-Net (U-Net++ [19]) have been widely used in medical image segmentation. In this paper, a multi-point supervised training network (MPS-Net) is proposed, which is based on U-Net network structure with multi-scale feature extraction structure, sieve connection structure and multi-point supervised structure, and it is used for automatic and accurate segmentation of COVID-19 CT images. The experimental results show that this method has better performance and certain competitiveness than the existing classical methods. The main contributions of this paper are as follows:

1. We propose a network model with a sieve connection (SC) structure, a multi-scale input structure and a multi-point supervised training structure for COVID-19 lesion segmentation.
2. Due to the geometrical features of the lesions are diverse, we use the sieve connection module to screen out the characteristic information of lesions of different sizes.
3. A multi-point supervised training structure is proposed to improve the segmentation accuracy of the model by using different scales ground truth at different up-sampling points.

The rest of this paper is organized as follows: Section II presents the related works; Section III presents the improved method; Section IV analyzes and discusses the experimental results; Section V summarizes the paper and draws our conclusions.

## II. Related Works

With the advances in artificial intelligence, many deep learning network models have been proposed for medical image processing. For example, Milletari *et al.* [20] proposed a U-Net-based V-Net for prostate MRI image segmentation network. V-Net combined with different MRI images to achieve end-to-end prostate segmentation. Wu *et al.* [21] introduced a COVID-19 classification and segmentation system, that was trained on a dataset containing 144,167 CT scans, collected from 400 COVID-19 patients and 350 uninfected cases. Their JCS model achieved a 78.3% Dice Coefficient on the segmentation test set, and a sensitivity of 95.0%, and a specificity of 93.0% on the classification test set. Jin *et al.* [22] created that DUNet network introduced the idea of transformable convolution based on U-Net. The algorithm utilizes the local characteristics of retinal vessels to achieve the end-to-end segmentation task. DUNet can adaptively adjust the size of the convolution kernel according to the thickness and shape of the segmented vessels, and obtain accurate vascular segmentation results based on multi-scale convolution. Recently, with the COVID-19 outbreak, some researchers have proposed segmentation models of COVID-19 lesions and achieved good performance. For example, Fan *et al.* [23] have proposed a new COVID-19 lung infection segmentation network (Inf-Net) that automatically segment the infected area. The Inf-Net uses a parallel partial decoder to generate a global map and aggregate high-level features. Then use explicit edge attention and implicit reverse attention to enhance the representation. In addition, the author also uses weak supervision to train the network. The dataset consists of 50 CT images with ground truth labels and 1600 CT images with pseudo labels. The DSC, sensitivity and specificity of the model were 0.739, 0.725 and 0.960 respectively. Zheng *et al.* [24] designed a multi-scale discriminant network (MSD-Net) for multi-class segmentation of COVID-19 lung infections on CT images. The network increases the receptive field through pyramid convolution block (PCB) to enhance the segmentation ability of infected areas of different sizes, and then uses channel attention block (CAB) structure and residual refinement block (RRB) structure to fuse and improve the feature map. The dice similarity coefficient (DSC), sensitivity and specificity of the model for three different types of infection were (0.7422, 0.8593, 0.9742), (0.7388, 0.8268, 0.9869) and (0.8769, 0.8645, 0.9889). Wang *et al.* [25] proposed a COPLE-Net automatic segmentation network and a new loss function (NR-Dice). It is characterized by noise robustness. The DSC of experimental results was 80.72±9.96. Xie *et al.* [26] proposed the RTSU network based on U-Net. A new non - local neural network (NNN) module is introduced to make use of the structured relationship. The proposed module learns the visual and geometric relations between all convolution features in order to generate self-attention weights and use transfer learning to conduct secondary training of the network. The results showed that RTSU-Net performed better than the other three models (3D-UNet, FRV-Net, PDV-Net) in severe pulmonary infection caused by COVID-19. Song *et al.* [27] used ResNet50 network to process all sections in each 3D chest CT image, and then formed the final prediction of each CT image. Wang *et al.* [28] combined U-Net and 3D-CNN for the diagnosis and assessment of COVID-19. The sensitivity and specificity of the DSC obtained by the model are 0.754, 0.972 and 0.922, respectively. Zhou et al [29] proposed a deep learning algorithm for solving the problem of large scenes and small objects. The algorithm decomposes the 3D segmentation problem into three 2D segmentation problems, thus reducing the complexity of the model by an order of magnitude. After several times of removing the poor quality sample images, the DSC of the algorithm segmentation results reached 0.903.

This paper presents an MPS-Net for CT image segmentation of COVID-19 lesion areas. We used the image enhancement method of rotation, flip, translation and cropping to expand 300 CT images to 9000 for network training, and 68 CT images were used to test the model after training. Dice similarity coefficient (DSC), Sensitivity (Sens), Specificity (Spec) and IOU were used to evaluate the segmentation results of the model. The Datasets from: https://medicalsegmentation.com/covid19.

## III. Method

U-Net is a convolutional neural network for biomedical image segmentation proposed by Ronneberger *et al.* in 2015 [18]. The network structure is composed of symmetrical encoder part on the left and decoder part on the right. The encoder part is used to extract features from the image, and the structure follows the typical structure of convolutional network. Each convolution layer contains two $3\times 3$ convolution operations, and each convolution operation is followed by an activation function (ReLu) and a max-pooling operation with a pooling size of $2\times 2$ and step size of 2 for down-sampling. The number of repetitions is four. In each down-sampling step, we double the number of feature channels. Decoders, on the other hand, are used to construct segmentation map based on features obtained from encoders. It consists of a $2\times 2$ transpose convolution of for up-sampling (feature channel reduced by half), which is connected to the encoder’s feature map and two $3\times 3$ convolutions, each followed similarly by a activation function ReLU. In the last layer, the classifier, $1\times 1$ convolution and a sigmoid activation function are used to generate the probability map of the final segmentation.

### A. Network Architecture

**FIGURE 2.**
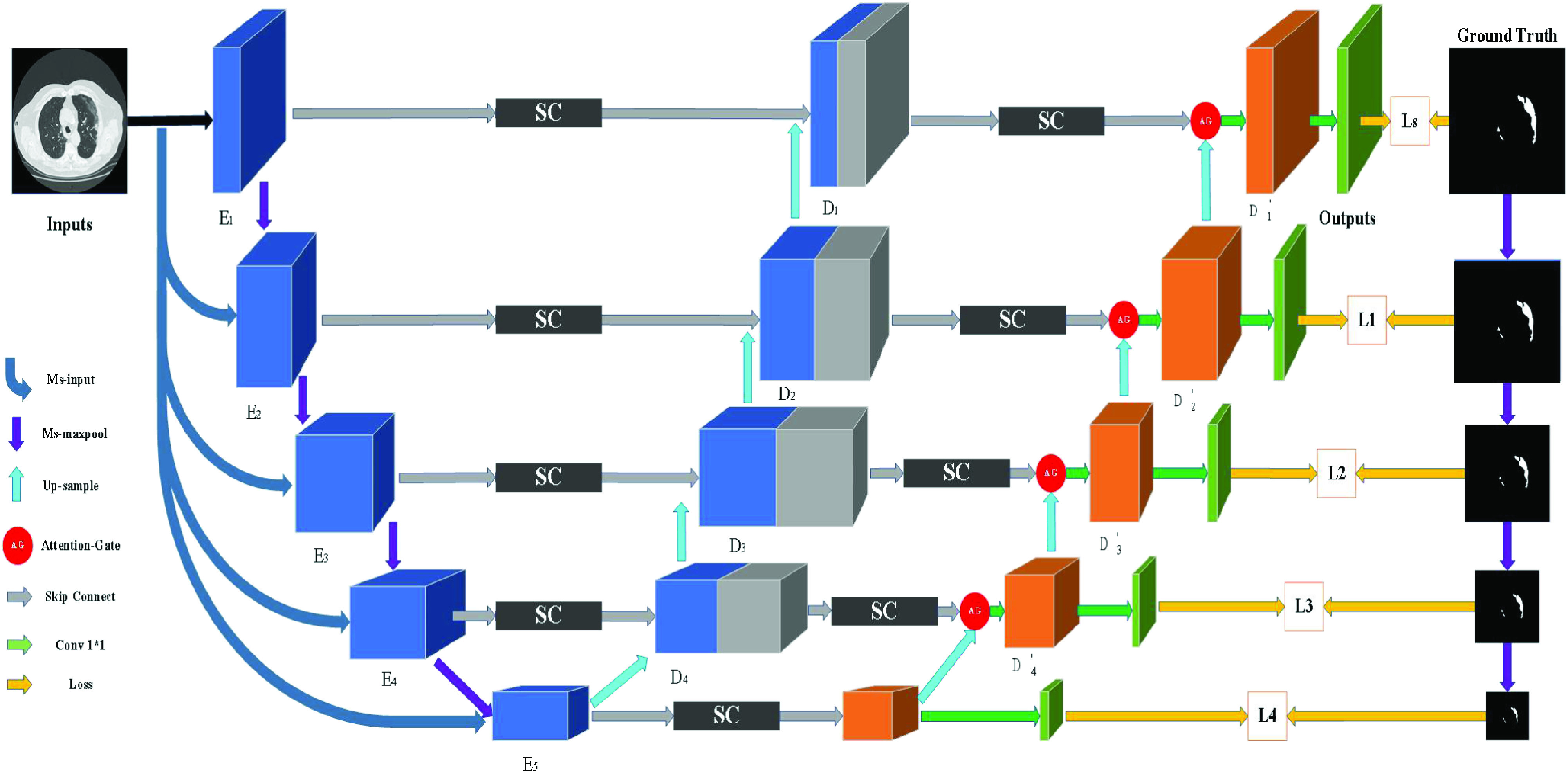
MPS-Net structure.

The classical U-Net model realizes the fusion of low-level image features and high-level image features through the multi-jump structure, so as to extract more features. In this paper, a new network based on U-Net is proposed for lesion segmentation of COVID-19 CT images. Multi-scale feature extraction structure is used to replace the $3\times 3$ convolution operation in U-Net. The multi-scale feature extraction module based on Inception structure adopts convolution of different kernel sizes to improve the generalization and expression ability of the network. Multi-scale input structure can compensate for lost edge position information in convolution and pooling operations. A sieve connection structure is designed to replace the original connection structure in U-Net and reduce the number of channels by half. At the same time, in order to preserve the location information of the target feature accurately, the maximum pool index is used in the upsampling process of the feature fusion decoder. The structure can retain the information of different granularity and ensure the comprehensiveness of the extracted information. In the decoder phase, We add second stage upsampling to extract more feature information. At first, we use the attention gate module to make the model pay more attention to the really important information in the training. Then, multi-point supervised training is introduced to improve the training accuracy of the model. The overall structure is shown in Fig.2.

### B. Multi-Scale Feature Extraction Modules

**FIGURE 3.**
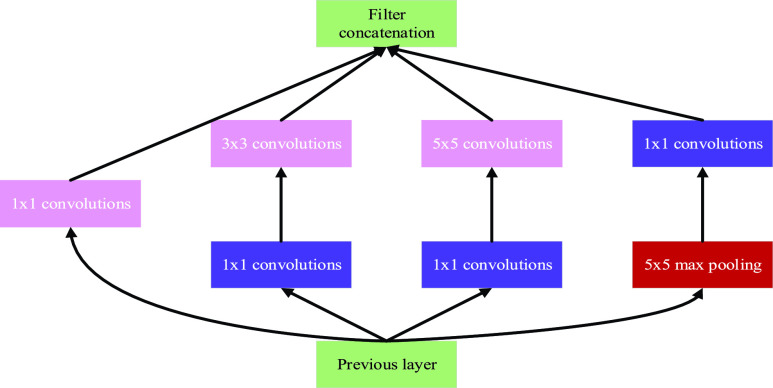
The basic inception module.

The Inception module [30] is the core structure of the GoogleNet network model that achieved the best results in ILSVRPC 2014. The structure of Inception network increases the depth and width of the network and improves the utilization of computing resources in the network while keeping the calculation budget unchanged. Multiple scale feature information is processed at the same layer using filters of different sizes, and then aggregated at the next layer in order to extract multiple scale fusion features in the next Inception module [31]. As shown in Fig.3, filters of sizes $1\times 1$, $3\times 3$, and $5\times 5$ are used for base Inception. The output of convolution and the max pool is concatenated, forming the input for the next stage. Rather than using a properly sized filter at one level, using multiple sized filters makes the network wider and deeper, so it can recognize different scale features. In order to further refine the feature, $1\times 1$ convolution is applied to reduce the feature dimension before the convolution of $3\times 3$ and $5\times5$.

**FIGURE 4.**
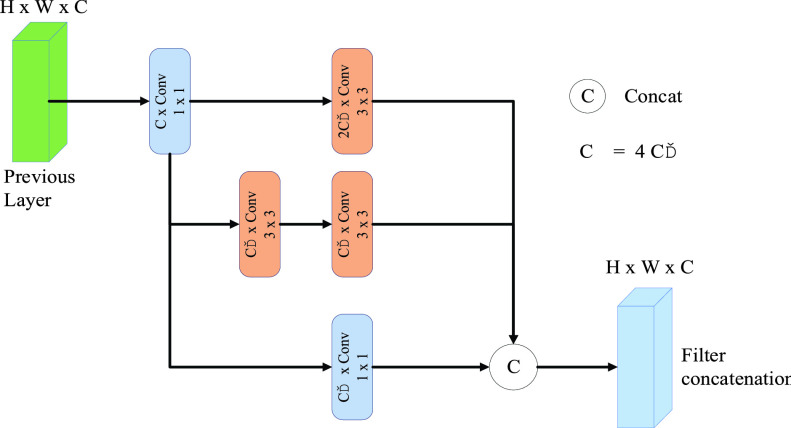
Multi-scale feature exception method based on an inception structure.

In this work, we use two $3\times 3$ filters in series instead of $5\times 5$ convolution filters, because they have an equivalent receiving field. This reduces the cost of calculation, resulting

in fewer parameters and less training time. The multi-scale feature extraction method based on inception structure is shown in Fig.4. Three sets of convolution operations ( $1\times 1$ convolution, $3\times 3$ convolution and two $3\times 3$ convolutions) with different scales of step size of 2 and padding of 0 are adopted in the previous layer feature map (H $\times $ W $\times $ C). After connecting the three sets of obtained feature maps, an activation function (ReLU) and a batch normalization (BN) are adopted.

The problem of overfitting can be alleviated by using BN layer. The convolution operation of multi-scale can make the network model have good ability to learn the target features of different scales.

### C. Sieve Connection Module

**FIGURE 5.**
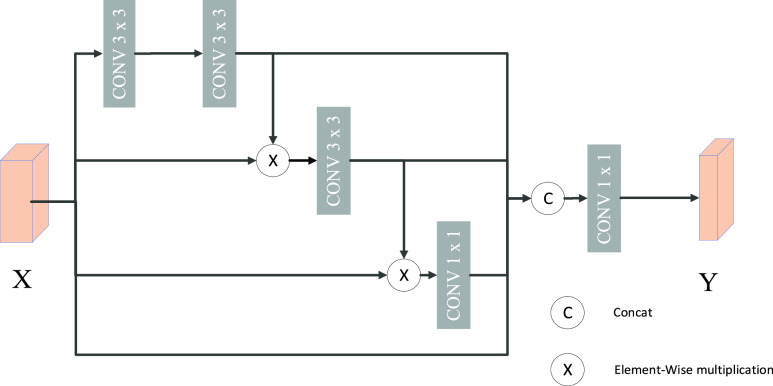
Sieve connection structure.

The sieve connection module (SCM) is used to replace the original skip connection layer in U-Net network. Similar to the multi-scale feature extraction structure, the sieve connection structure also adopts three different size convolution kernels. When the convolution operation with reduced receptive field was used, the input feature map is elements-wise multiplied by the feature graph with larger receptive field as input. Then, the extracted feature maps with three different scales are combined with the input feature maps, and the convolution of $1\times 1$ is used to alleviate the semantic gap between the low-level features and the high-level features, and the number of feature channels is reduced by half. The sieve skip connection structure is shown in Fig.5.

### D. Multi-Scale Input Model

Convolution and pooling inevitably lead to the loss of pixel position information, which can be alleviated by using multi-scale input (image pyramid). An image pyramid is a collection derived from the same original image and arranges in a pyramid shape with progressively reduced resolution. The bottom of the pyramid is a high-resolution representation of the image to be processed, while the top is a low-resolution approximation.

**FIGURE 6.**
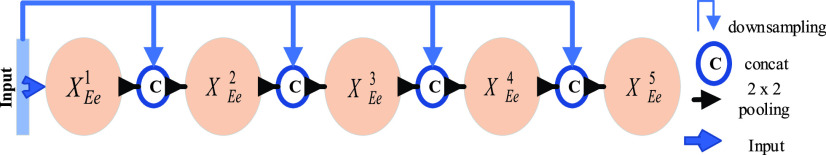
Multi-Scale Input Model.

In this paper, the input image is downsampled with a size of 2 and a step size of 2 according to the steps of the encoder, followed by the convolution operation of $3\times 3$. Then, the feature map obtained from the multi-scale input and the output feature map obtained from the lower sampling of the upper layer are concatenated as the input feature of this layer, as shown in Fig.6.

### E. The Decoding Module

The decoding module is composed of attention module and multi-point supervision training module. In the existing methods, the single signal supervised training model is mostly used. To improve the accuracy of segmentation results, we designed a multi-point supervision (MPS) training module. On the basis of U-Net structure, the module introduces the second stage of up-sampling to further extract the feature information. After each up-sampling, the attention gates (AGs) is used to make the network pay more attention to the important information. AGs are commonly used for natural image analysis, knowledge graphs, and language processing (NLP), for image subtitle [32], machine translation [33], [34], and classification [35]–[37] tasks. The initial work is to explore the attention map by interpreting the gradient of the output category score relative to the input image.

**FIGURE 7.**
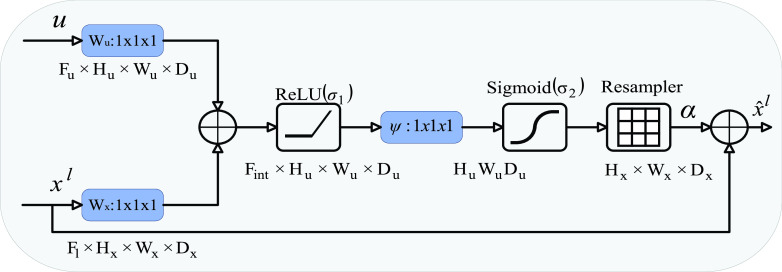
Attention Gate structure.

It has also been used for image classification and image segmentation in recent years [38]. The schematic diagram of the structure is shown in the Fig.7. Input features (Xl) are Scaled with attention coefficients ( $\alpha$) computed in AG. Spatial regions are selected by analysing both the activations and contextual information provided by the gating signal (g) which is collected from a coarser scale. Grid resampling of attention coefficients is done using trilinear interpolation. At the same time, the information extracted in the first stage of up-sampling is used in the attention module to eliminate the ambiguity of irrelevant and noise response in the second sieve skip connection and highlight the significant characteristics of sieve skip connection transmission. The convolution operation of size $1\times 1$ is used to reduce the channel number of the feature graph obtained by AG to 2. The obtained feature image and the downsampling gold standard image are used for multi-point training. The experimental results show that the multi-point supervised network module can effectively improve the segmentation accuracy of the network. The structure diagram is shown in Fig.2.

### F. Loss Function

Early pneumonia lesions often occupy a small area of the image. If cross-entropy is used for training, the predicted results may deviate significantly from the background. The Dice loss function proposed by Milletari *et al.* [20] can effectively avoid this problem, and the formula is as follows:

\begin{equation*} Loss_{\textrm {Dice}} =1-\frac {2\sum \nolimits _{i}^{N} {p_{i} g_{i}} }{\sum \nolimits _{i}^{N} p_{i}^{2} +\sum \nolimits _{i}^{N} g_{i}^{2} }=\frac {\sum \nolimits _{i}^{N} {(p_{i} -g_{i})^{2}}}{\sum \nolimits _{i}^{N} p _{i}^{2} +\sum \nolimits _{i}^{N} g_{i}^{2}}\tag{1}\end{equation*}

where $N$ is the number of pixels in the image, $p_{i}$ is the probability predicted by the neural network, and $g_{i}$ is the pixel value corresponding to the gold standard. In addition, according to the formula, when the Dice coefficient is close to 1, the prediction result will be close to the ground true value. However, since the use of the Dice loss function will lead to a lower recall rate of the segmentation results, according to the Tversky index [39], we use the Tversky loss function on the basis of Dice. The definition of the loss function is as follows:

\begin{align*} Loss_{Tversky} =\frac {\sum \nolimits _{i=1}^{N} {p_{0i} g_{0i}} }{\sum \nolimits _{i=1}^{N} {p_{0i} g_{0i}} +\alpha \sum \nolimits _{i=1}^{N} {p_{0i} g_{1i}} +\beta \sum \nolimits _{i=1}^{N} {p_{1i} g_{0i}}}\!\!\!\! \\ {}\tag{2}\end{align*}

where, $\text{p}_{\mathrm {0i}}$ is the probability that the output is predicted to be pathological, and $\text{p}_{\mathrm {1i}}$ is the probability that the output is predicted to be non-pathological. Similarly, $\text{g}_{\mathrm {0i}}$ is 1 and $\text{g}_{\mathrm {1i}}$ is 0 for the lesion area, and $\text{g}_{\mathrm {0i}}$ is 0 and $\text{g}_{\mathrm {1i}}$ is 1 for the lesion area. By adjusting the hyperparameters $\alpha $ and $\beta $, we can control the tradeoff between false positives and false negatives. When $\alpha = \beta =0.5$, this function becomes the Dice loss function. When $\alpha = \beta =1$, the function becomes the Jaccard loss function. In this paper, we set $\alpha $ to 0.4 and $\beta $ to 0.6.

Binary cross entropy (bce) is a special case of cross-entropy Loss function and is used only for dichotomous problems, and the formula is as follows:

\begin{align*} Loss_{binary\_{}cross\_{}entropy} =-\sum {\left [{ {y~~1-y} }\right] \left [{ {\begin{array}{l} \log \left ({p }\right) \\ \log \left ({{1-p} }\right) \\ \end{array}} }\right]} \\ {}\tag{3}\end{align*}

where, y and p were ground truth and prediction, y was 0 or 1, and p was between 0 and 1. Therefore, the loss function adopted by the network is defined as:

\begin{equation*} Loss=\lambda Loss_{bce} +\left ({{1-\lambda } }\right)Loss_{Tversky}\tag{4}\end{equation*}

where, $\lambda $ is the penalty coefficient used to balance the two functions and takes the value of 0.6.

Since the multi-point supervision model is adopted in this paper and 5 supervisory signals are extracted in the decoding stage, the overall loss function of this model is defined as follows:

\begin{equation*} Loss_{overall} =\alpha Ls+\sum \limits _{i} {\beta _{i} L_{i}}\tag{5}\end{equation*}

where, Ls and Li respectively represent loss functions Ls, L1, L2, L3 and L4 in FIG. 2, and their formulae are uniformly defined as (4). $\alpha $ is 0.6, $\beta $ is 0.4.

### G. Experiment Setup

We used a PC equipped with an Intel Core i7-10700k, 3.8 GHz CPU with 16 GB RAM and 8 GB of RTX2070S GPU for MSFFU-Net training. The operating system of the computer was 64-bit Win10. The structure of the network was implemented under the open source deep learning library TensorFlow with Pycharm implementation. In addition, Numpy (scientific computing library), some methods of image processing in OpenCV and some libraries in sklearn were used for image processing. All the COVID-19 CT images in our experiments had been resized to $512\times512$.

In the process of model training, the stochastic gradient descent optimization algorithm was used to iteratively solve the parameters of the COVID-9 image segmentation network. The initial learning rate was set to 0.0001, which was changed to 0.1 times the current value every 20 epochs. The batch size was set to 2, total training epochs to 50, and the order of the data set was randomly scrambled after each training epoch. It took about 80h to train the network on this platform.

### H. Evaluation Metrics

In order to quantitatively evaluate the performance of the segmentation results of the proposed algorithm, four evaluation indicators were used in this paper named: Sensitivity (Sens), Specificity (Spec), IOU and DSC to evaluate the experimental results. Sensitivity is the ratio of the number of correctly detected COVID-19 infection pixels to the total number of detected pixels. Specificity is the ratio of the number of correctly detected non-COVID-19 infection pixels to the total number of non-COVID-19 infection pixels. Dice is the ratio of twice the number of correctly detected COVID-19 infection to the sum of the total number of detected pixels and the total number of ground truth pixels. The expressions of Sens, Spec, IOU and DSC are defined as follows:

\begin{align*} Sensitivity=&\frac {TP}{TP+FN} \tag{6}\\ Specificity=&\frac {TN}{FP+TN} \tag{7}\\ DSC(G,S)=&\frac {2\left |{ {G\cap S} }\right |}{\left |{ G }\right | + \left |{ S }\right |} \tag{8}\\ IOU(G,S)=&\frac {\left |{ {G\cap S} }\right |}{\left |{ G }\right |\cup \left |{ S }\right |}\tag{9}\end{align*}

where *TP, TN, FP, FN, G,* and $S$ denote true positive, true negative, false positive, false negative, ground truth and segmentation, respectively. In this model, positive refers to COVID-19 infection and negative refers to background. Therefore, they are the four kinds of COVID-19 infection segmentation results based on the fact that each pixel can be segmented correctly or incorrectly. TP represents infection pixels are correctly detected as COVID-19 infection; TN represents background pixels are correctly detected as background; FP represents background pixels are incorrectly detected as COVID-19 infection; FN represents infection pixels are incorrectly detected as background.

## IV. Results and Discussion

**FIGURE 8.**
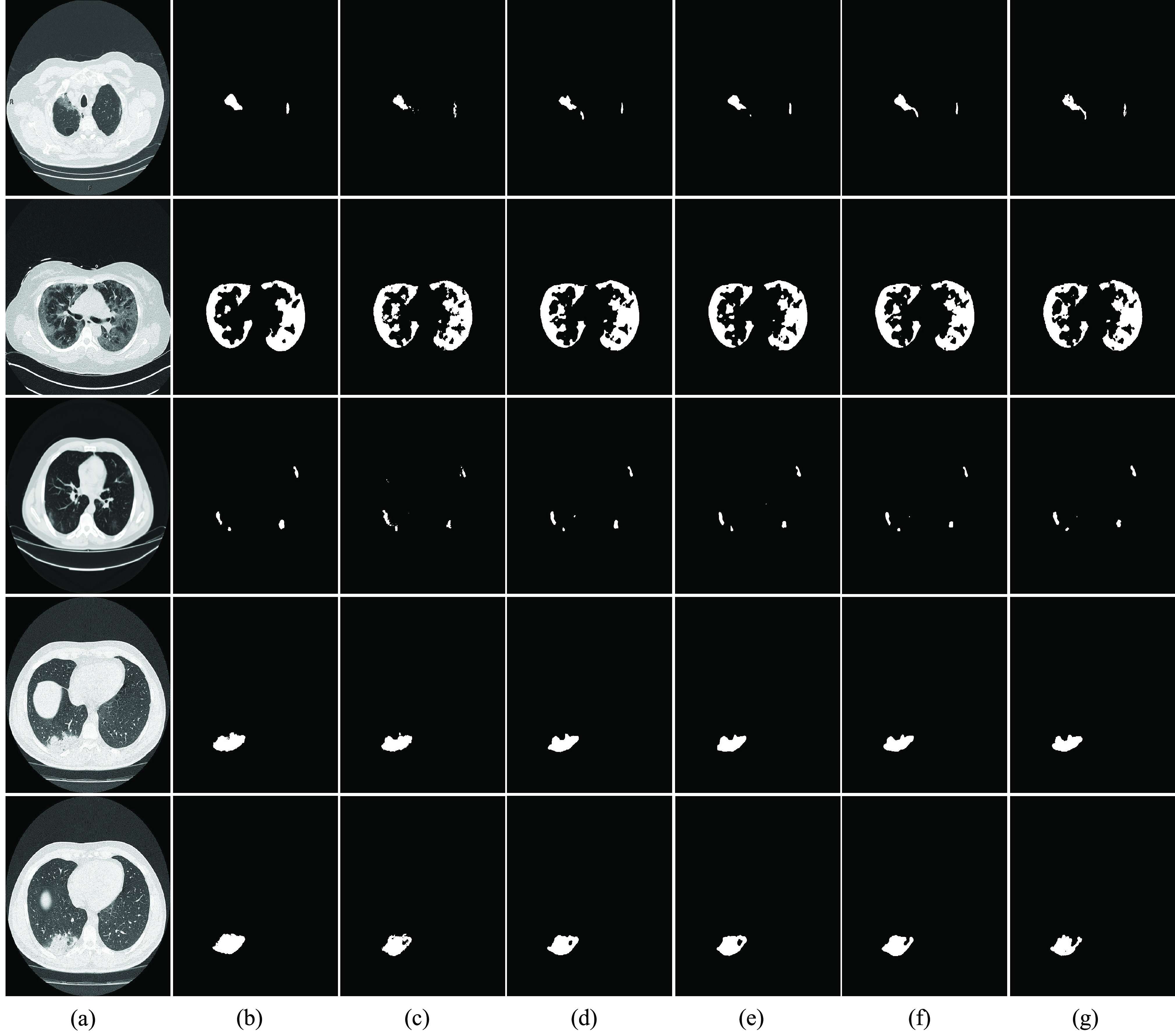
Visualization results of COVID-19 lesion segmentation. (a) The original image. (b) Segmentation with U-Net. (c) Segmentation with H-DenseUnet. (d) Segmentation with U-Net++. (e) Segmentation with MPS-Net (Without multi-point supervision). (f) Segmentation with MPS-Net. (g) Ground-truth segmentation.

Segmentation results of COVID-19 images are shown in Fig.8. In order to verify the performance of the model, we compared three models with our model and used the same parameters, configuration environment and training sets. Table 1 presents DSC, Sens., Spec and IOU results for different models. Compared with U-Net, H-DenseUNet, and Unet++, DSC scores increased by 8%, 4.1% and 1.1%, respectively. Sens scores increased by 3.6%, 5.6% and 0.4%, respectively; IOU scores improved by 9.4%, 6% and 1.3%, respectively. Compared to our own model, after adding multi-point monitoring, Dice scores, Sens scores, Spec scores and IOU scores increased by 1.7%, 2% and 1.7%, respectively.

**TABLE 1:** Comparison With Other Methods

Method	MPS Model	Dice.	Sens.	Spec.	IOU
U-Net		0.7525	0.8061	0.9977	0.646
H-DenseUnet		0.7910	0.7841	0.9987	0.689
U-Net++		0.8216	0.8366	0.9987	0.729
MPS-Net		0.8159	0.8205	0.9987	0.725
	✓	0.8325	0.8406	0.9988	0.742

Fig.8 shows a qualitative comparison between our model and the other four segmentation models. COVID-19 lesions showed rich diversity in morphology and area. This property leads to segmentation difficulties. Remarkably, the MSP-Net proposed in this paper can effectively solve this problem, indicate that our MSP-Net outperform the baseline methods. Specifically, they produce segmentation results that are close to the basic facts, and far fewer organizations are missegmented. In Fig.8, column (a) represents the original CT image; Column (b) represents the segmentation results of V-Net; (c) Column represents the segmentation results of the H-DenseUNet; Column (d) represents the segmentation result of U-Net ++; Columns (e) represents the segmentation result of MSP-Net without multi-point supervision; Columns (f) and (g) represent the segmentation results of MSP-Net and the ground truth, respectively.

**FIGURE 9.**
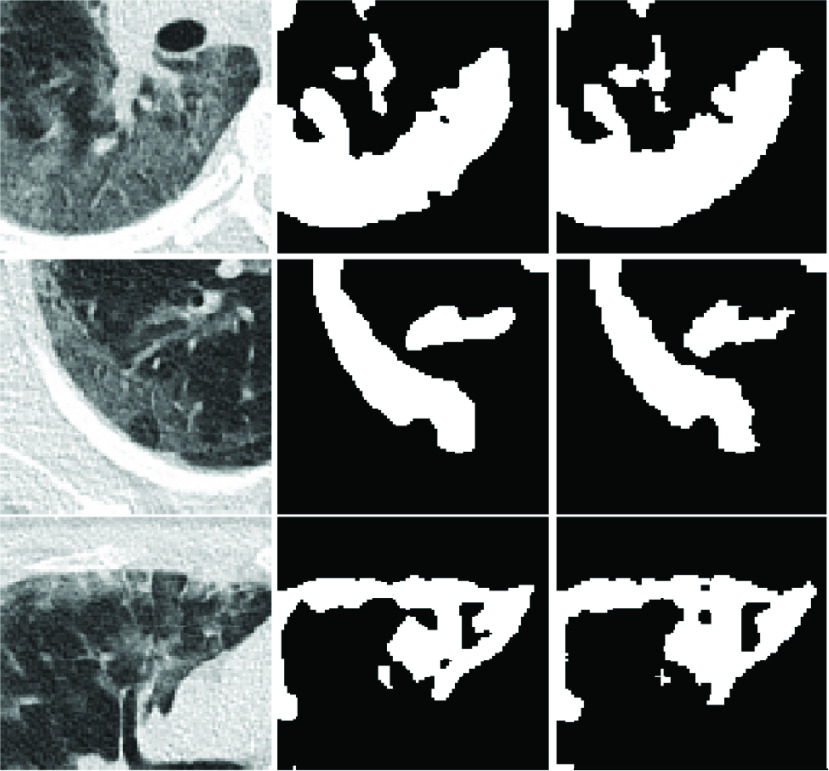
COVID-19 images segmentation local examples. From left to right: (a) Original images. (b) Groundtruth images. (c) Segmentation results.

Fig.9 shows the detailed information of segmentation results more clearly. It can be seen that the method proposed in this paper can accurately segment the pathological areas of COVID-19 CT images. It has low over-segmentation rate and missing segmentation rate.

**FIGURE 10.**
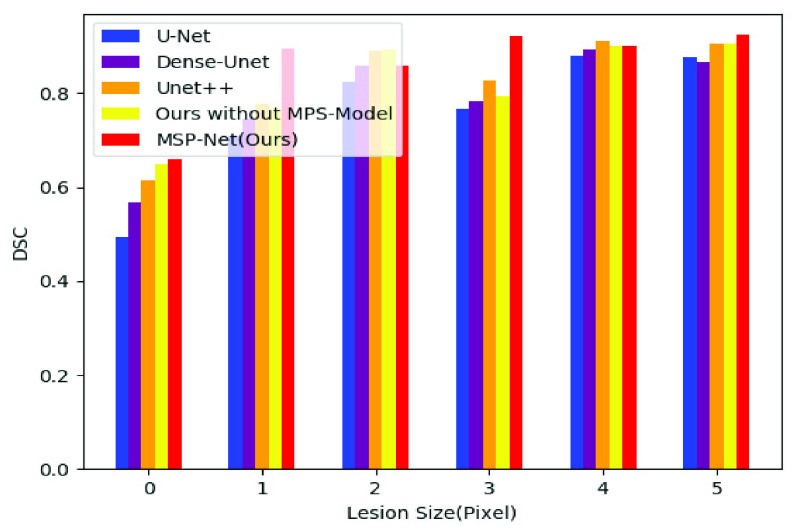
DSC results of different models in six groups of samples of different sizes.

**FIGURE 11.**
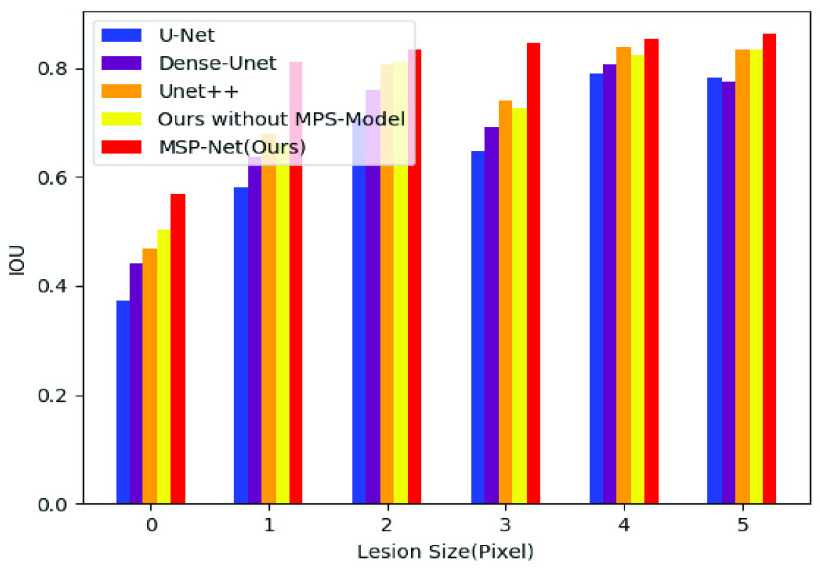
IOU results of different models in six groups of samples of different sizes.

In order to further verify the segmentation effect of our model, we divided the test samples into six parts according to the size of lesion area (0–1K, 1K–2K, 2K–3K, 3K–4K, 4–5K, >= 5K, the unit is the number of pixels) and evaluate the segmentation results after using different models to segment these parts. Fig.10 shows the DSC scores after the segmentation of the six groups of samples using different models. As shown in the figure, the method proposed in this paper is superior to other methods in all the six groups of samples and significantly improves the segmentation accuracy of samples of different sizes. Fig.11 shows the IOU scores after the segmentation of the six groups of samples using different models. The data distribution is shown in Table 2. And the best results are highlighted.

**TABLE 2:** Compare the Dice and IOU of Different Groups

Model	DSC	IOU	0–1k	1k–2k	2k–3k	3k–4k	4k–5k	>5k
U-Net	✓		0.494	0.709	0.825	0.768	0.881	0.876
		✓	0.373	0.582	0.705	0.648	0.790	0.782
H-DenseUnet	✓		0.568	0.747	0.891	0.784	0.892	0.867
		✓	0.441	0.678	0.805	0.692	0.807	0.775
U-Net++	✓		0.614	0.778	0.926	0.827	0.911	0.906
		✓	0.467	0.678	0.805	0.741	0.837	0.832
MPS-Net (Without MPS-model)	✓		0.648	0.768	0.893	0.792	0.901	0.906
		✓	0.503	0.666	0.810	0.725	0.823	0.833
MPS-Net(ours)	✓		0.659	0.896	0.858	0.922	0.901	0.924
		✓	0.539	0.813	0.773	0.856	0.807	0.863

**FIGURE 12.**
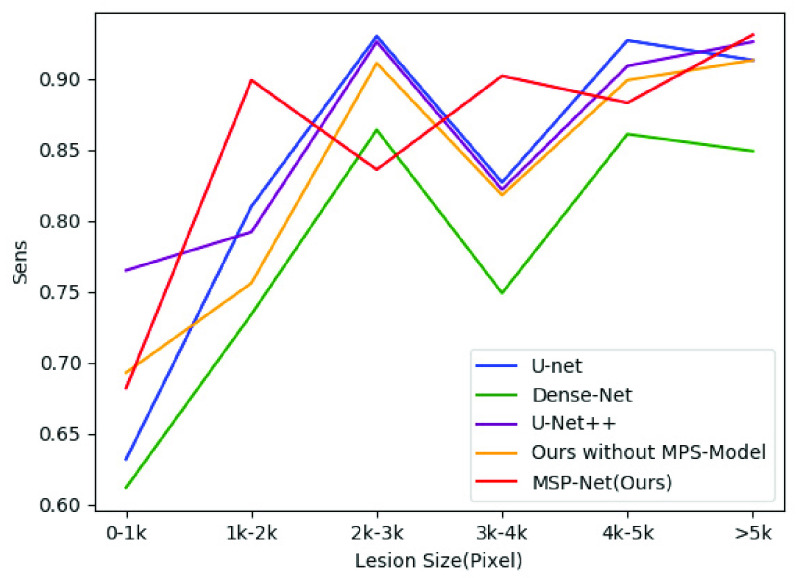
Sensitivity results of different models in six sets of samples of different sizes.

**FIGURE 13.**
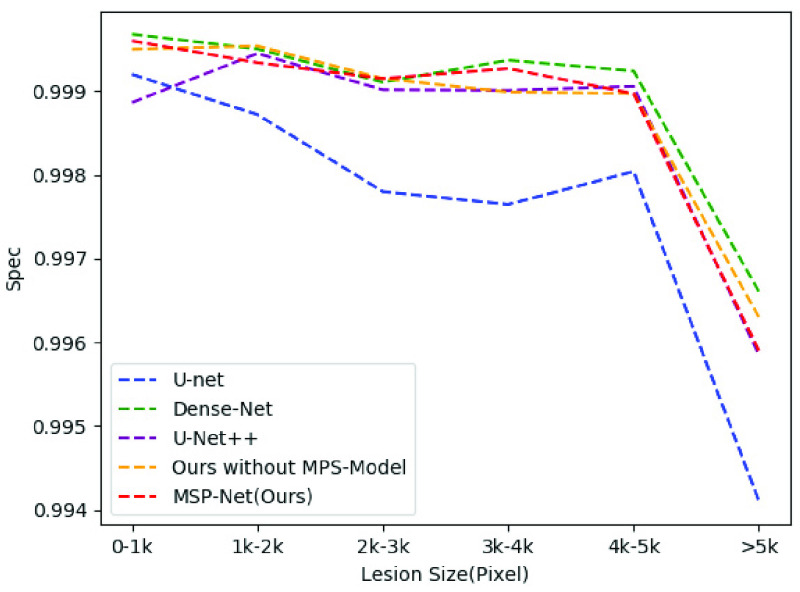
Specificity results of different models in six sets of samples of different sizes.

Fig.12 shows the Sens scores after the segmentation of these six groups of samples using different models. Fig.13 shows the Spec scores after the segmentation of these six groups of samples using different models. As the figure shows, our model is overall superior to the others. The data distribution is shown in Table 3. And the best results are highlighted.

**TABLE 3:** Compare the Sens and Spec of Different Groups

Model	Sens	Spec	0–1k	1k–2k	2k–3k	3k–4k	4k–5k	>5k
U-Net	✓		0.632	0.810	0.930	0.827	0.927	0.913
		✓	0.999	0.998	0.997	0.997	0.998	0.994
H-DenseUNet	✓		0.612	0.734	0.864	0.749	0.861	0.849
		✓	0.999	0.999	0.999	0.999	0.999	0.996
U-Net++	✓		0.765	0.792	0.926	0.822	0.909	0.926
		✓	0.998	0.999	0.999	0.999	0.999	0.995
MPS-Net (Without MPS-model)	✓		0.693	0.756	0.911	0.818	0.899	0.913
		✓	0.999	0.999	0.999	0.998	0.998	0.996
MPS-Net(ours)	✓		0.682	0.899	0.836	0.902	0.883	0.931
		✓	0.999	0.999	0.999	0.999	0.997	0.996

Through qualitative and quantitative analysis results, it can be seen that our MSD-Net is superior to other segmentation models and can produce more accurate segmentation results, which are closer to the real situation and less misorganization of segmentation.

## V. Conclusion

Under the circumstance that novel coronavirus has not been effectively controlled in the world and medical resources are effective, it is of great significance to use deep learning to assist doctors in clinical diagnosis. In clinical diagnosis, the geometrical shape of the lesion plays an important role in the correct diagnosis. Therefore, the correct segmentation of lesions can effectively help doctors to make a correct diagnosis. In this paper, we propose a multi-point supervised network for segmentation of COVID-19 lesions. We introduced the multi-scale feature extraction module on the basis of U-net network, replaced the original skip connection structure of U-Net with the sieve connection, reduced the boundary missing caused by convolution operation through the multi-scale input model, and introduced the multi-point supervision structure to improve the segmentation accuracy. Compared with other advanced network models, our network has better segmentation results. DSC index, Sens index, Spec index and IOU index are 0.8325, 0.8406, 0.9988 and 0.742, respectively. The experimental results show that the proposed MPS-Net can effectively segment the COVID-19 CT infected lesions.
